# Correlates of delayed sexual intercourse and condom use among adolescents in Uganda: a cross-sectional study

**DOI:** 10.1186/1471-2458-12-817

**Published:** 2012-09-21

**Authors:** Liesbeth E Rijsdijk, Arjan ER Bos, Rico Lie, Robert AC Ruiter, Joanne N Leerlooijer, Gerjo Kok

**Affiliations:** 1 Windesheim University of Applied Sciences, Windesheim Honours College, Zwolle, the Netherlands; 2 Open University, Heerlen, the Netherlands; 3 Wageningen University, Wageningen, the Netherlands; 4 Maastricht University, Maastricht, the Netherlands; 5 RutgersWPF, Utrecht, the Netherlands

**Keywords:** Ugandan adolescents, Delayed sexual intercourse, Condom use, Attitudes, Social norms, Self-efficacy, Segmented approach, sub-Saharan Africa

## Abstract

**Background:**

Comprehensive sex education, including the promotion of consistent condom use, is still an important intervention strategy in tackling unplanned pregnancies, HIV/AIDS and sexually transmitted infections (STIs) among Ugandan adolescents. This study examines predictors of the intention to use a condom and the intention to delay sexual intercourse among secondary school students (aged 12–20) in Uganda.

**Methods:**

A school-based sample was drawn from 48 secondary schools throughout Uganda. Participants (*N* = 1978) completed a survey in English measuring beliefs regarding pregnancy, STIs and HIV and AIDS, attitudes, social norms and self-efficacy towards condom use and abstinence/delay, intention to use a condom and intention to delay sexual intercourse. As secondary sexual abstinence is one of the recommended ways for preventing HIV, STIs and unplanned pregnancies among the sexually experienced, participants with and without previous sexual experience were compared.

**Results:**

For adolescents without sexual experience (virgins), self-efficacy, perceived social norms and attitude towards condom use predicted the intention to use condoms. Among those with sexual experience (non-virgins), only perceived social norm was a significant predictor. The intention to delay sexual intercourse was, however, predicted similarly for both groups, with attitudes, perceived social norm and self-efficacy being significant predictors.

**Conclusions:**

This study has established relevant predictors of intentions of safe sex among young Ugandans and has shown that the intention to use condoms is motivated by different factors depending on previous sexual experience. A segmented approach to intervention development and implementation is thus recommended.

## Background

In contrast to most sub-Saharan African countries, Uganda reported a dramatic decline in HIV prevalence, from about 18% in 1992 [1] to about 4.1% in 2003 [2]. However, HIV prevalence has since risen and is now stabilized at around 7.0% [3]. Recent research and trend analyses show an increase in risk-taking behaviours, particularly in multiple sexual partnership and non-spousal sex [4]. A decrease in condom use in non-spousal sex among men has also been noted [1].

Almost 14% of Ugandan adolescents is sexually active by age 15 (15.5% of the girls and 12.2% of the boys) [3] and over 50% by age 18 [5]. However, in a study among sexually active unmarried Ugandans aged 15 to 24, it was concluded that after initiating sexual intercourse, some adolescents decide to abstain from sex until they are older, married, or have a paid job [6]. Nearly two thirds of the sexually active respondents abstained for three months, slightly less than half abstained for six months and 18% abstained for twelve months. Taking alcohol, going to parties and talking to parents about sex, were found as associating factors with discontinuation of secondary abstinence. Secondary abstinence is more common in sub-Saharan Africa than in other regions of the world [7]. When sexually active, around 15% of the young Ugandans aged 15–19 years, ever used a condom. Of those aged 15–24 years who ever had sex, 28% used a condom at first sex, with a slight difference between boys (29%) and girls (27%). By the age of 19, approximately 50% of all Ugandan women have had their first child [5]. Moreover, 36% of the births among Ugandan adolescents mothers younger than 20 years are unintended [8].

These figures show that comprehensive sex education, including the promotion of consistent condom use, is still an important intervention strategy in tackling unplanned pregnancies, HIV/AIDS and sexually transmitted infections (STIs) among Ugandan adolescents. This is also recognised by the Ugandan government, as young people are included as one of the main target groups for prevention efforts in Uganda [9].

Comprehensive sex education programmes are likely to be most effective when they are based upon theory- and evidence- based needs assessments and intervention strategies [10-12]. Hence it is important to know which factors determine safe sexual behaviour, i.e., condom use and (secondary) delay of sexual intercourse.

### Determinants of condom use and delay of sexual intercourse

As safe sex behaviour requires (individual) planning and forethought, socio-cognitive theories like the Theory of Planned Behaviour (TPB) [13,14], and, more recently, its successor the Reasoned Action Approach (RAA) [15], are often used to design interventions that promote safe sex behaviour. According to the RAA, the most important determinant of planned behaviour is the intention to perform the behaviour. This is supported by a meta-analysis of 47 experimental tests of intention-behaviour relations which showed that a medium to large sized change in intention leads to a small to medium sized change in behaviour [16]. Behavioural intention, in turn, is the result of a combination of attitudes towards the behaviour, the perceived norm (e.g. descriptive and injunctive norms, social encouragement, pressure) and self-efficacy [14,15,17]. Attitude is defined as a person’s disposition to respond favourably or unfavourably towards a certain behaviour. The perceived norm is a function of beliefs that specific, important individuals or groups (e.g. friends, parents, one’s girlfriend, one’s husband) approve or disapprove of a certain behaviour as well as beliefs that these referents themselves perform or do not perform that specific behaviour. Self-efficacy, also termed perceived behavioural control, refers to “the extent to which people believe that they are capable of, or have control over, performing a given behaviour” [15:155].

The RAA has been supported by research, mainly conducted in the USA and Western Europe, on the adoption of many health-related behaviours [18,19], including condom use [20-26] and sexual intercourse delay [13,27]. Although unsafe sexual behaviour is an important health risk among Sub-Saharan African adolescents [28], there have only been a few recent studies on the socio-cognitive determinants of (the intention of) delaying sexual intercourse and (the intention of) condom use in an African setting that meet international criteria concerning sample size and the use of multivariate statistical methods. Nonetheless, despite the paucity of research, the studies that have been conducted appear to support RAA; notably on correlates and determinants of condom use [11,29-33]; on both primary abstinence and condom use [34] and on sexual activity and condom use intentions [35]. To the best of our knowledge, there is only one study on secondary abstinence and its determinants [6], but this study does not consider socio-cognitive determinants.

Studies combining primary delay, secondary abstinence and condom use are, to the best of our knowledge, non-existent. When researching the correlates and predictors of these three main preventive safe sex behaviours among sub-Saharan adolescents, the previous sexual experience of participants should be considered. In fact, on the basis of their systematic review of school-based sexual health interventions to prevent STI and HIV in sub-Saharan Africa, Paul-Ebhohimhen, Poobalan, and van Teijlingen [36] concluded that behaviour change related to either abstinence or condom use is greatly influenced by participants’ pre-intervention sexual history (i.e. having previously been sexually active or not). This clearly suggests that the determinants of, and explained variance for, (secondary) sexual intercourse delay and condom use could differ between adolescents who, at baseline, have already engaged in (anal or vaginal) sexual intercourse and adolescents who have not.

This study sets out to determine the socio-cognitive correlates and key predictors of the intention to use a condom the next time one has sex and the intention to (primary or secondary) delay sexual intercourse among young people in Uganda. Furthermore, it sought to assess differences in predictors between adolescents with and without previous sexual experience.

## Method

### Participants

Data were derived from a cross-sectional survey conducted with secondary school students (*N* = 1986), aged 12–20 years, in Uganda. A total of 48 schools were selected throughout Uganda. Some schools were segregated by gender, others were not. Also, some schools were day schools while others were boarding schools. At all schools, the principal or a teacher approached 50 students to participate in the study. To ensure an equal distribution of gender and age, the principals/teachers were asked to select a group of 50 students on the basis of gender (if possible) and age. Participation in the study was on a voluntary basis. In total, 887 boys and 1099 girls participated, yielding a response rate of 83%. The mean age of the participants was 16.1 years (*SD* = 1.87). Eight participants (0.4%; 2 boys and 6 girls) were subsequently excluded from analyses as they specifically indicated they were raped. The final sample was *N* = 1978 (885 boys and 1093 girls).

### Procedure

Data collection took place in March of 2008. The participants completed a self-administered questionnaire in English in their classroom under the supervision of two research assistants who ensured that the room was quiet and that participants did not discuss their answers with others. They also provided clarification on questionnaire items if necessary. The research assistants were first year undergraduate students at the Makerere University in Kampala, Uganda and were, prior to data collection, trained for three days by two researchers, one Ugandan and one Dutch, on the objectives of the study, the structure of and rationale for the questionnaire, how to best respond to participants’ questions about the questionnaire (e.g., What is meant by anal sexual intercourse?), and how to go about creating and maintaining a safe, quiet environment in which participants could anonymously complete their questionnaire. After completing the questionnaire, participants were given a soft drink and a snack as well as a Health Referral List with information on counselling services in their district, if available. All participants provided informed consent and the Ethics Committee at Maastricht University’s Faculty of Psychology and Neuroscience in the Netherlands approved this study prior to initiation.

The questionnaire was derived from a questionnaire previously used in Tanzania and South Africa [37] and pre-tested among adolescents from a school in Kampala. The pre-test yielded minor adaptations to the questionnaire. Two versions of the questionnaire were developed: one for boys and one for girls. Both contained the same questions but different wording (e.g., girlfriend versus boyfriend).

### Variables and measurements

The dependent variables were the intention to delay sexual intercourse and the intention to use a condom the next time one engages in sexual intercourse. The independent variables were derived from the RAA and included attitudes, perceived social norms and self-efficacy towards delaying sexual intercourse and towards condom use. Additionally, items relating to age, gender, beliefs about pregnancy, HIV/AIDS and STI and sexual experience (having had sexual intercourse or not) were included. Pearson’s correlations were assessed for constructs consisting of two variables at a *p* < .01 level. Mean scores for items measuring a similar underlying construct (scales) were employed. For some constructs, the correlation was not significant, in which case we used separate items. Most items were measured using 5-point Likert scales. If measured otherwise, it will be indicated in the text.

Intention to delay sexual intercourse was measured by the following item: “Do you think you will wait to have sexual intercourse until you are older?” Attitude towards delayed sexual intercourse was measured by two items (*r* = .31; *p* < .01): “It is better that young people my age who are in a steady relationship postpone sexual intercourse until they are older” and “Young people my age should not engage in sex until they are older”. Perceived social norm for delayed sexual intercourse was measured by two items (*r* = .22; *p* < .01): “My friends believe that it is acceptable for people my age to have sexual intercourse with a steady boyfriend/girlfriend” and “My friends believe that people my age should postpone sexual intercourse until they are older.”). Self-efficacy to delay sexual intercourse was measured by two items (*r* = .45; *p* < .01).: “For me, waiting to have sexual intercourse until I am older is difficult” and “I am confident that I can wait to have sexual intercourse until I am older”.

Intention to use a condom was measured by the following item: “Do you think you and your (future) lover will use a condom when you have sexual intercourse?”. Attitude towards condom use was measured by two separate items: “It is wise to use a condom during sexual intercourse” and “It is pleasant to use a condom”. Perceived social norm for condom use was measured by one item: “My friends think that people my age should use a condom when having sexual intercourse”. Self-efficacy for condom use was measured by two items (*r* = .32; *p* < .01) : “For me, using a condom every time I have sexual intercourse is difficult” and “I am sure I can use a condom every time I have sexual intercourse”.

Beliefs about pregnancy were measured by two items (*r* = .44; *p* < .01): “A girl cannot get pregnant the first time she has sex” and “If a girl washes herself thoroughly immediately after sexual intercourse, she cannot get pregnant”. Beliefs about HIV/AIDS were measured by the following two statements (*r* = .16; *p* < .01).: “HIV can be transmitted through mosquito bites” and “When someone is HIV-infected, this can always be seen in their appearance”*.* Beliefs about STI were measured by two statements (*r* = .22; *p* < .01): “Biologically, boys have a higher risk of contracting sexually transmitted infections than girls” and “Anal sexual intercourse is a safe way to protect oneself from sexually transmitted infections”.

To assess whether respondents were sexually experienced or not, they were asked two questions: “With how many different people have you had sexual intercourse in your life?” (None; with … people; I don’t want to talk about this; I don’t know), and: “Have you ever practised anal sexual intercourse? (No; Yes; I don’t want to talk about this). Those who indicated they had sexual intercourse (including ‘anal’ sexual intercourse) before had to answer a third set of questions, relating to their first and last sexual experience (e.g. age at first sex, how long ago they last had sexual intercourse and their experience at first and last sex (e.g. forced; tricked; consensual). Those respondents who answered “None” and “no” to the first two questions, and skipped the questions meant for those who had had sexual intercourse before, were defined as “without sexual experience”. All remaining respondents who answered the questions meant for those who had had sexual intercourse before, were defined as “sexual experienced”, unless they answered these questions inconsistently. Those participants were defined as “unknown”.

### Data analysis

Means, standard deviations, frequencies and percentages were calculated to provide a description of the sexual history of the sample. Bivariate correlations between the intention to use condoms and the intention to delay sexual intercourse, on the one hand, and the relevant determinants, on the other, were then assessed. Subsequently, linear regression was employed to determine the relative importance of the correlates of the intention to use condoms and the intention to delay sexual intercourse. A p-value of .01 was used at a 95% confidence interval. All variables in our model for sexual intercourse delay and in our model for condom use had low levels of collinearity. For delayed sexual intercourse, the variance inflation factor [VIF] was 1.19 and 1.15 among participants with and without previous sexual experience, respectively. For condom use, the VIF was 1.15 and 1.12. The tolerance level for all models was > .2. Data were analysed using SPSS 17.0.

## Results

### Socio-demographic and sexual experience of the respondents

Table 1 shows the demographic (age, gender) and sexual experience of the respondents. The mean age of the respondents was 16.1 years (SD = 1.86). Of the total sample, 55.3% (885) were boys and 44.7% (1093) were girls. More than a quarter (27.4%) of the respondents indicated they had engaged in sexual intercourse: 37.2% of the boys (N = 885), and 19.4% of the girls (N = 1093), of which 10.5% reported having had anal sexual intercourse (96 boys and 62 girls). Girls indicated more often than boys that they did not have any sexual experience (i.e. vaginal or anal intercourse): 58.4% of the girls against 36.6% of the boys. The previous sexual experience of the remaining participants (24%) is unknown as these participants did not answer the items measuring past sexual behaviour or answered these questions inconsistently.

**Table 1:** Demographic and sexual history profile of the respondents

		**Sexually experienced**	**Not sexually experienced**	**Unknown**	**Total (*N*)**
Male	= < 12	16.7%	50.0%	33.3%	12
	13-14	24.1%	42.4%	33.5%	158
	15-16	36.2%	36.6%	27.2%	298
	17-18	42.8%	36.7%	20.5%	278
	= > 19	44.6%	28.8%	26.6%	139
	Male total	37.2%	36.6%	26.2%	885
Female	= < 12	15.8%	57.9%	26.3%	19
	13-14	10.0%	65.7%	24.3%	239
	15-16	17.3%	61.1%	21.6%	468
	17-18	28.7%	50.2%	21.2%	293
	= > 19	27.0%	50.0%	23.0%	74
	Female total	19.4%	58.4%	22.2%	1093
All participants	= < 12	16.1%	54.8%	29.0%	31
	13-14	15.6%	56.4%	28.0%	397
	15-16	24.7%	51.6%	23.8%	766
	17-18	35.6%	43.6%	20.8%	571
	= > 19	38.5%	36.2%	25.4%	213
	Total	541	962	475	1978

### Delayed sexual intercourse: correlations with socio-cognitive factors

Table 2 shows means, standard deviations and inter-correlations between the intention to delay sexual intercourse and the various socio-cognitive factors. The mean scores of intention to delay intercourse, beliefs about pregnancy, beliefs about HIV, attitude towards delayed sexual intercourse, self-efficacy to delay intercourse and the perceived social norm were just below or just over 4 (e.g. agree), which is relatively high. The intention to delay sexual intercourse was positively correlated with beliefs about pregnancy (*r* = .14; *p* < .01), beliefs about STI (*r* = .09; *p* < .01), beliefs about HIV (*r* = .09; *p* < .01), attitude towards delayed sexual intercourse (*r* = .36; *p* < .01), self-efficacy to delay sexual intercourse (*r* = .46; *p* < .01) and the perceived social norm for delayed sexual intercourse (*r* = .07; *p* < .01).

**Table 2:** Means, standard deviations and intercorrelations of the determinants of the intention to delay sexual intercourse

**Variable**	**M (SD)**	**1**	**2**	**3**	**4**	**5**	**6**	**7**
1. Intention to delay sexual intercourse	3.98 (1.28)	-	14*	.09*	.09*	.36*	.46*	.07*
2. Beliefs about pregnancy	3.78 (.99)		-	.36*	.33*	.14*	.22*	.03
3. Beliefs about HIV	3.57 (.97)			-	.30*	.10*	.12*	.01
4. Beliefs about STI	3.25 (.96)				-	.13*	.18*	.01
5. Attitude	4.11 (.96)					-	.36*	.18*
6. Perceived social norm	3.65 (1.35)						-	.06
7. Self-efficacy	3.97 (1.04)							-

*N* = 1978; *p < .01 (two tailed).

### Condom use: correlations with socio-cognitive factors

Table 3 shows means, standard deviations and inter-correlations between the intention to use a condom and the various socio-cognitive factors. Intention to use a condom, beliefs about pregnancy, beliefs about HIV, attitude towards condom use (both it is wise to use a condom and it is pleasant to use a condom) and the perceived social norm scored relatively high (> 3.5). The intention to use condoms was positively correlated with both attitude items, namely ‘it is wise to use a condom’ (*r* = .15; *p* < .01), and ‘it is pleasant to use a condom’ (*r* = .08; *p* < .01). Intention to use condoms was also positively correlated with self-efficacy for condom use (*r* = .29; *p* < .01) and the perceived social norm towards condom use (*r* = .18; *p* < .01).

**Table 3:** Means, standard deviations and intercorrelations of the determinants of the intention to use condoms

**Variable**	**M (SD)**	**1**	**2**	**3**	**4**	**5**	**6**	**7**	**8**
1. Intention condom use	3.79 (1.30)	-	.05	.02	.04	.15*	.08*	.29*	.18*
2. Beliefs about pregnancy	3.78 (.99)		-	.36*	.33*	.05	-.13*	.07*	.00
3. Beliefs about HIV	3.57 (.97)			-	30*	.04	-.07	.05	.02
4. Belief s about STI	3.25 (.96)				-	.04	-.10*	.04	.01
5. Attitude to condom use as wise	3.94 (1.20)					-	.16*	.18*	.23*
6.Attitude to condom use as pleasant	3.52 (1.29)						-	.14*	.16*
7.Perceived social norm	3.82 (1.22)							-	.19*
8. Self-efficacy	3.33 (1.04)								-

*N* = 1978; *p < .01 (two tailed).

### Predictors of intention to delay sexual intercourse and secondary abstinence

To assess the relative importance of the predictors of the intention to delay sexual intercourse, linear regression analyses were conducted. Table 4 shows the main results of the linear regression model predicting the intention to delay sexual intercourse. For participants without previous sexual experience, age (*β* = −.07; *p* < .05) was negatively related to the intention to delay intercourse, thus showing that the older the participant is, the less likely it is that he or she intends to delay sexual intercourse. Furthermore, beliefs about pregnancy (*β* = .07, *p* < .05), attitudes towards delaying sexual intercourse (*β* = .13, *p* < .001), the perceived social norms (*β* = .15, *p* < .001), and self-efficacy for delayed sexual intercourse (*β* = .30, *p* < .001) were positively associated with the intention to delay sexual intercourse. Together, these predictors explained 23% of the variance for adolescents without previous sexual experience.

**Table 4:** Main results of linear regression analyses predicting the intention to delay

	**Intention Delay**		**Intention Delay**	
	**No sexual experience (N = 962)**		**Sexual experience (N = 541)**	
	*R*^2^	*β*	*R*^2^	*β*
Step 1	.23		.21	
1. Age		-.07*		-.02
2. Beliefs about pregnancy		.07*		-.05
3. Beliefs about HIV		.06		.03
4. Beliefs about STI		.01		.00
5. Attitudes towards delay		.13***		.18***
6. perceived social norm		.15***		.15**
7. Self-efficacy		.30***		.30***

*p < .05; **p < .01; ***p < .001.

Among participants with previous sexual experience, the intention to secondary abstinence was positively related to attitude towards delayed intercourse (*β* = .18, *p* < .001), the perceived social norm towards delayed sexual intercourse (*β* = .15, *p* < .01) and self-efficacy to delay intercourse (*β* = .30, *p* < .001). Together, these predictors explained 21% of the variance for adolescents with previous sexual experience.

### Predictors of intention to use condoms

Table 5 shows the main results of the linear regression analyses predicting the intention to use a condom. Among participants without previous sexual experience, the attitude that using condoms is wise (*β* = .11; *p* < .01), the perceived social norm towards condom use (*β* = .10; *p* < .01) and self-efficacy to use condoms (*β* = .33; *p* < .001) were positively associated with the intention to use a condom, and, together, explained 17% of the variance in the intention to use a condom. Among participants with previous sexual experience, the perceived social norm was the only correlate (*β* = .14, *p* < .01), explaining 7% of the variance in the intention to use a condom among this group.

**Table 5:** Main results of linear regression analyses predicting the intention to condom use

	**Intention to use condoms**		**Intention to use condoms**	
	**No sexual experience (N = 962)**		**Sexual experience (N = 541)**	
	*R*^2^	*β*	*R*^2^	*β*
Step 1	.17		.07	
1. Age		-.03		.06
2. Beliefs about pregnancy		-.04		.11
3. Beliefs about HIV		.01		.00
4. Beliefs about STI		.04		-.07
5. Attitude to condom use as being wise		.11**		-.02
6. Attitude to condom use as being pleasant		-.03		.07
7. Perceived social norm		.10**		.14**
8. Self-efficacy		.33***		.09

*p < .05; **p < .01; ***p < .001.

## Discussion

The present study examined, among adolescents in Uganda, the predictors of the intention to delay sexual intercourse (for virgins), the intention to secondary abstinence (for non-virgins) and the intention to use a condom the next time one has sex. This study has shown that when it comes to the intention to delay sexual intercourse, compared to the intention to secondary abstinence, the predictors are relatively similar. For both groups (virgins and non-virgins), attitudes towards, perceived social norms regarding and self-efficacy to delay sexual intercourse were significant predictors of the intention to delay sexual intercourse. Additionally, among those without previous sexual experience, age was negatively related to the intention to delay intercourse thus implying that the likelihood that adolescents delay sexual intercourse decreases with age. Furthermore, among adolescents without previous sexual experience, beliefs about pregnancy were positively related to the intention to delay sexual intercourse. In sub-Saharan Africa, including Uganda, “secondary abstinence” is common among young people [6,38-40]. This might explain the similarity in predictors of delayed sexual intercourse between participants with and participants without previous sexual experience.

With respect to the predictors of the intention to use condoms, our findings demonstrate a clear difference between adolescents with and adolescents without previous sexual experience. Among those without previous experience, attitudes towards condom use, the perceived social norm regarding condom use and self-efficacy to use a condom were important predictors of the intention to use a condom. Among those with previous sexual experience, only the perceived social norm appeared to predict intention to use a condom. With this group, self-efficacy and attitude were non-significant.

Overall, our findings support, at least in part, the RAA as a means of understanding what underlies the intention to delay sexual intercourse, secondary abstinence and the intention to use condoms among sub-Saharan African adolescents. However, the variance explained by the predictors (.23 for the intention to delay sexual intercourse and .17 for the intention to use a condom) was lower than what is normally found in studies conducted in Europe and the USA. On average, RAA factors explain about 39% of variance in intention to perform a certain behaviour in European and American contexts [19,20,22]. African studies tend to show less explained variance. In fact, of the few studies conducted in an African context, only one demonstrated an explained variance comparable to studies conducted in Europe and the USA [11]. In that study, not only socio-cognitive factors but also geographic variables, socio-economic factors and access to condoms were included as predictors of condom use. In line with Fishbein [17], Ajzen [13], and supported by the fact that the explained variance in our study is different from that in other studies conducted in Europe and the USA, we contend that the relative importance of each of the RAA variables may vary according to the behaviour, the population targeted and the socio-cultural context in which that behaviour takes place.

Further, in our study, we found that, among adolescents with previous sexual experience, only the perceived social norm predicted condom use. This is supported by other studies conducted in an African context, where subjective norms were found to be (one of) the strongest predictors of condom use [30,41,42]. This dominant role of perceived norm may be, at least in part, attributable to the more collectivist nature of African societies in contrast to the more individualistic decision-making that occurs in Europe and North America [43,44].

Although this study has demonstrated that there are important socio-cognitive predictors of delayed sexual intercourse and condom use, we must recognize that actual delay in sexual intercourse and actual condom use is also impacted by environmental factors (e.g., access to condoms) [45,46], structural factors (e.g., poverty and legal context) [47,48], and one’s ability (e.g., skills necessary to discuss delayed sexual intercourse or use condoms) [49,50]. Additionally, socio-cultural factors, like gender roles that impact the degree to which girls can discuss condom use or buy and carry condoms, the presence of HIV-related stigma, and taboos on discussing sexuality in general, are all negatively related to condom use [51,52].

### Strengths and limitations

This study is one of the few to assess the socio-cognitive predictors of delayed sexual intercourse and condom use among a large sample of young people in a sub-Saharan African context. Furthermore, it is, to our knowledge, the first study to explore the influence of previous sexual experience on the intention to use condoms and (secondary) delayed sexual intercourse. As such, it contributes to filling the gap in the current literature on the determinants of safe sex behaviour among adolescents in low-income countries where HIV and AIDS are major health risks for young people, and where secondary abstinence is more common than in other parts of the world [53]. Furthermore, knowing that the determinants of safe sex behaviour differ for those with and those without previous sexual experience provides valuable input for the development of safe sex interventions among Ugandan adolescents.

This study also has some limitations. First, the questionnaire was in English, which for some participants may have been difficult to comprehend. Problems with comprehension were dealt with by training research assistants to provide vernacular translations for texts that students found difficult to understand. Second, the questionnaires were administered in classrooms where privacy was not always possible. Despite having given research assistants careful instructions to secure privacy and confidentiality, this could not always be guaranteed (e.g. teacher walking around the classroom). Third, the Principles of the schools were asked to select the participants based on an equal distribution of gender (if possible) and age. This non-random selection may lead to biased results, as the Principals may have selected those students who are “positive” role-models. Fourth, this study investigated only socio-cognitive factors. It is possible that other factors such as environmental factors (e.g. availability of affordable condoms), structural factors (poverty and legal context), factors related to ability (e.g. being able to discuss delayed sexual intercourse or condom use) and socio-cultural factors (e.g. gender norms, HIV-related stigma and taboos on talking about sexuality) could explain some variance in the intention to delay sexual intercourse, intention to secondary abstinence and the intention to use condoms. Fifth, because we used cross-sectional data, we were only able to assess associations between variables. We could not draw conclusions regarding causality. Sixth, in our study, we did not measure actual sexual intercourse delay and actual condom use. We relied solely on the fact that other studies have demonstrated a robust relationship between intention and actual behaviour [16,22,31]. Seventh, the respondents were all secondary school students, which limit the conclusions made to this group only. This fact should be taken into account when interpreting the results of this study, as adolescents who are not attending school may be more vulnerable to early sexual debut, HIV and other sexually transmitted infections and unintended pregnancy [54]. Finally, we assessed the socio-cognitive factors using single items or two-items constructs. This could be seen as a possible shortcoming. However, it has previously been argued that the use of single items or two-item constructs is viable if the item correctly measures the core element of the construct [55]. We did indeed seek to construct our single-item and two-item measures in such a way that they represented the central aspect of the construct being measured.

Future research should employ a longitudinal research design thereby enabling causal inferences regarding the impact of the determinants of delayed sexual intercourse and condom use on the actual behaviour using scales that are reliable in a sub-Saharan African context.

## Conclusions

In conclusion, this study has improved our understanding of the socio-cognitive predictors of the intention to delayed sexual intercourse, to secondary abstinence and condom use among Ugandan adolescents with and without previous sexual experience. Our findings have several implications for the development of comprehensive sex education programmes geared to adolescents in Uganda.

Firstly, the RAA has proven to be a useful framework for assessing correlates of safe sex behaviour, also in the Ugandan context. We therefore suggest that comprehensive sex education programmes geared to young Ugandans address the socio-cognitive predictors found in this study, namely attitudes towards, social norms about and self-efficacy to delay intercourse among both adolescents with and adolescents without previous sexual experience, and, for adolescents with previous sexual experience, social norms towards condom use. For adolescents without previous sexual experience, attitudes towards, social norms about and self-efficacy to use condoms must also be addressed.

Secondly, a segmented approach based on actual previous sexual experience is important in order to take into account the differences between adolescents with and adolescents without previous sexual experience. As the likelihood of sexual activity increases with age, we contend that it would be wise to start comprehensive sex education before adolescents start experimenting with sexual intercourse. This contention is reinforced by previous research demonstrating that it is easier to change socio-cognitive variables and promote actual safe sex behaviour among adolescents who are not yet sexually active than among those who are [56]. We recommend that such programmes address attitudes, social norms and self-efficacy to delay sexual intercourse and to use condoms. From about the age of 15 on, sex education should focus on (perceived) social norms, i.e., emphasizing that important others (peers) want to delay sexual intercourse, do not want to feel pressured to have sex, and do intend to use a condom when they do decide to have sex. This can be done using role models and peer education [57], as peer-educators are considered to be credible sources of information by the adolescent target group [58], who are more likely to understand their situation [59], and who can support young people in developing positive group norms and in making healthy decisions about sex [57,60]. A prerequisite for using peer-education as a strategy is that the peer educators are adequately trained and well supported by professional health educators to be able to educate their peers effectively [57,60].

Also, facilitating an open discussion and interaction among young people could be a useful strategy as it would enable them to hear what their peers really think and (intend to) do and because it may encourage them to form their own opinions and norms.

Finally, as sex education programmes are most likely to be effective when they are theory and evidence based, intervention development should be rooted in a context-specific needs assessment that assesses, among other things, specific religious, economic, political and cultural factors that may influence safe sex behaviour [12]. As such, a needs assessment in the Ugandan context should explore the environmental constraints (e.g. access to condoms), abilities (e.g. skills to discuss delayed sexual intercourse or use condoms) and socio-economic and cultural factors (e.g. gender norms concerning sexuality, stigma and taboos, poverty leading to risky sexual behaviour, collectivism) present in a sub-Saharan context.

## Competing interests

The authors declare that they have no competing interests.

## Authors' contributions

LER participated in the design and the overall coordination of the study, conducted the analysis and drafted the manuscript. AERB, RL, RACR and JNL participated in the design of the study, checked the analysis and contributed to the writing of the manuscript. GK and the other authors read and approved the final draft.

## Pre-publication history

The pre-publication history for this paper can be accessed here:

http://www.biomedcentral.com/1471-2458/12/817/prepub
